# AIOLOS-Associated Inborn Errors of Immunity

**DOI:** 10.1007/s10875-024-01730-9

**Published:** 2024-05-22

**Authors:** Motoi Yamashita, Tomohiro Morio

**Affiliations:** 1 Laboratory for Transcriptional Regulation, RIKEN Center for Integrative Medical Sciences, 1-7-22, Suehiro-cho, Tsurumi, Yokohama, Kanagawa 230-0045 Japan; 2 Department of Pediatrics and Developmental Biology, Graduate School of Medical and Dental Sciences, Tokyo Medical and Dental University (TMDU), 1-5-45 Yushima, Bunkyo, Tokyo 113-8519 Japan; 3 Laboratory of Immunology and Molecular Medicine, Advanced Research Institute, Tokyo Medical and Dental University (TMDU), 1-5-45 Yushima, Bunkyo, Tokyo 113-8519 Japan

**Keywords:** AIOLOS, *IKZF3*, IKZF, IKAROS, Inborn errors of immunity

## Abstract

AIOLOS, encoded by the *IKZF3* gene, belongs to the Ikaros zinc finger transcription factor family and plays a pivotal role in regulating lymphocyte development. Recently, heterozygous missense loss-of-function variants within the DNA-binding domain of the *IKZF3* gene (G159R, N160S, and G191R) have been identified in patients with inborn errors of immunity (IEI). Additionally, a missense and a truncating variant (E82K and Q402X) leading to the AIOLOS haploinsufficiency have been documented. The majority of individuals with AIOLOS-associated IEI manifest recurrent sinopulmonary infections, as well as various bacterial and viral infections. The patients carrying the AIOLOS^N160S^ variant exhibit severe immunodeficient phenotypes. In contrast, patients harboring AIOLOS haploinsufficient variants predominantly present with clinical phenotypes associated with immune dysregulation. A varying degree of B-lymphopenia and hypoimmunoglobulinemia was noted in approximately half of the patients. Mouse models of AIOLOS^G159R^ and AIOLOS^N160S^ variants (Aiolos^G158R^ and Aiolos^N159S^ in mice, respectively) recapitulated most of the immune abnormalities observed in the patients. Among these models, Aiolos^G158R^ mice prominently exhibited defects in early B cell differentiation resulting from mutant Aiolos interfering with Ikaros function through heterodimer formation. In contrast, Aiolos^N159S^ mice did not manifest early B cell differentiation defects. However, they displayed a distinct immune abnormality characterized by impaired induction of CD62L expression in lymphocytes, which is likely attributable to dysfunction of Ikaros, leading to defective lymphocyte homing to lymph nodes. Considering the diverse clinical phenotypes observed in the reported cases and the distinct molecular pathogenesis associated with each variant, further studies with more patients with AIOLOS-associated IEI would contribute to a better understanding of the clinical spectrum and underlying molecular mechanisms associated with this disorder.

## Introduction

Determination of cell fate across a diverse array of cell types arises intrinsically from distinct gene expression profiles. These cell-type-specific gene expression patterns are shaped by the coordinated actions of various cell-type-specific transcription factors. In the context of human disorders related to the immune system, it has been widely established that perturbations in the activities of crucial transcription factors governing immune cell development contribute to the development of aberrant cells (cancers), defects in the immune system (immunodeficiency), or both. Inborn errors of immunity (IEI) include nearly 500 distinct inherited monogenic disorders that result in various disorders of the human immune system [1]. IEIs frequently manifest as exceedingly rare conditions, with only a limited cohort of documented patients. With bioengineering applications of CRISPR/Cas9 technology, it is now feasible to produce animal models that replicate the genetic variants identified in humans. The investigation of animal models of such diseases has yielded valuable insights into the underlying causes of rare IEIs and has facilitated the comprehension of the intricate molecular mechanisms driving their pathogenesis. In this article, we review clinical and immunological aspects of AIOLOS-associated IEI, as well as studies on its murine models to elucidate the fundamental molecular mechanisms that underlie this condition.

## AIOLOS in Lymphocyte Development

**Fig. 1:**
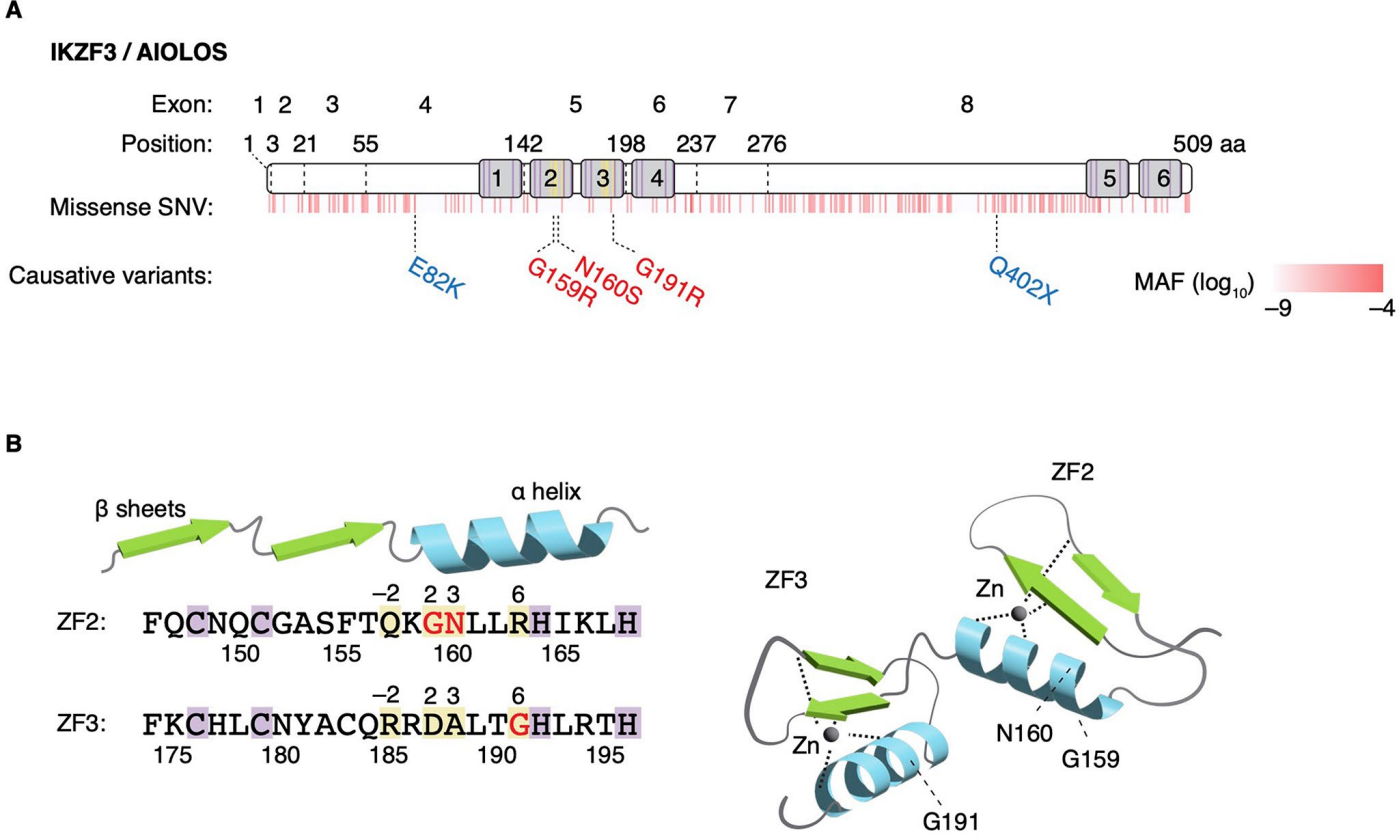
Structure of IKZF3/AIOLOS and IEI-causing variants. (**A**) The DNA-binding N-terminal ZFs (ZF1–4) and dimerizing C-terminal ZFs (ZF5–6) are denoted by gray boxes, with the positions of zinc-interacting cysteine and histidine residues highlighted by purple lines. The frequency of missense SNVs is represented in red at their corresponding positions. These SNV frequencies were sourced from the gnomAD database. ZF1–4 exhibit a notable scarcity of missense SNVs within the general population. IEI-causing AIOLOS variants are highlighted in red, denoting LOF and DN effect against KAROS and AIOLOS (G159R), or against AIOLOS alone (N160S and G191R). Haploinsufficient variants (E82K and Q402X) are represented in blue. (**B**) The amino acid sequence (left) and 3D structure (right) of AIOLOS ZF2 and ZF3 are shown. Positions − 2, 2, 3, and 6 relative to the initiation of the α-helix (highlighted by yellow shading) are established as critical for DNA binding. Specifically, Gly159 and Asn160 are at positions 2 and 3 in the ZF2 and Gly191 is at position 6, respectively, relative to the start of the α-helix. DN, dominant-negative; LOF, loss-of-function; MAF, minor allele frequency; SNV, single nucleotide variant; ZF, zinc finger

AIOLOS, encoded by the *IKZF3* gene, belongs to the Ikaros zinc finger (IKZF) transcription factor family, a group of proteins that play essential roles in regulating the development of lymphocytes and other hematopoietic cells. Aiolos was first cloned as a binding partner of Ikaros, the first IKZF molecule discovered [2]. Structurally, IKZF proteins contain four or three N-terminal zinc fingers (ZFs) that mediate DNA binding and two C-terminal ZFs that enable the homo- and heterodimerization of IKZF molecules (Fig. 1). IKZF proteins act as repressors or activators of their target genes. They interact with the transcriptional corepressor SIN3B and ATP-dependent nucleosome remodeler Mi-2β through ZFs, comprising a transcription factor complex also known as the nucleosome remodeling deacetylase complex [3–5]. IKZF proteins also interact with the SWItch/Sucrose Non-Fermentable (SWI/SNF)-related complex and polycomb repressive complex 2 (PRC2).

The role of Aiolos in lymphocyte development is well established in B cell differentiation. Although it is also expressed in T-lineage cells, its highest expression was observed in B-lineage cells [2]. During B cell development, Aiolos expression is induced during the transition from large pre-B cells to small pre-B cells by interferon regulatory factors (IRF) 4 and 8 [6]. Together with Ikaros, Aiolos downregulates the expression of the pre-B cell receptor (pre-BCR) components VpreB and Lambda5 as well as Myc expression by directly binding to the *Myc* promoter [6–8]. Consequently, large pre-B cells exit the proliferation state induced by pre-BCR signalling and undergo further development into the small pre-B cell stage. Aiolos sets the thresholds for BCR signaling in peripheral B cells to control B cell activation [9, 10]. The loss of Aiolos results in the breakdown of B cell tolerance, development of autoimmunity, and spontaneous autoantibody production [9].

Aiolos is also involved in T cell development. In the context of T helper (Th)17 cell development, Aiolos expression is induced by signal transducer and activator of transcription 3 (STAT3) and aryl hydrocarbon receptor (Ahr) in response to the interleukin (IL)-6 and transforming growth factor (TGF)-β [11]. Aiolos suppresses IL-2 expression, thereby promoting Th17 cell differentiation. Given that IL-2 plays a pivotal role in regulating the differentiation of various Th subsets such as Th1 cells, regulatory T cells (Treg), and follicular helper T cells (Tfh), Aiolos plays a regulatory role in the development of these Th subsets [12, 13]. The expression of Aiolos and Ikaros correlates with the Tfh lineage-defining factor B cell lymphoma (Bcl)-6 [14]. Ikaros, Aiolos, and Stat3 bind to the *Bcl6* promoter region to induce *Bcl6* expression. In addition, Aiolos positively regulates the expression of IL-10 in human CD4^+^ T cell populations [15, 16].

Aiolos plays a crucial role in the terminal maturation of natural killer (NK) cells [17]. Moreover, the targeted degradation of AIOLOS and IKAROS using lenalidomide led to a suppression of innate lymphoid cell (ILC)-1 and NK cell differentiation, whereas the function of ILC-3 was maintained [18].

## Human AIOLOS-Associated IEI

Genome-wide association studies have identified single nucleotide polymorphisms within the *IKZF3* locus that are associated with a range of diseases, including primary biliary cirrhosis [19], type I diabetes mellitus [20], and systemic lupus erythematosus [21]. Genomic variations resulting in the loss or functional degradation of AIOLOS are highly intolerant. Within the general population, the occurrence of alleles carrying stop-gain variants in the *IKZF3* gene is exceedingly rare, estimated to be at a frequency of approximately 3e-5 at maximum. *IKZF3* was not recognized as a causative gene for human monogenic diseases until two germline heterozygous missense variants in the second ZF of AIOLOS, G159R and N160S, were reported in patients with IEI [22, 23] (Fig. 1). Subsequent to these initial publications, additional heterozygous AIOLOS variants resulting in AIOLOS haploinsufficiency (E82K and Q402X) were recently elucidated [24]. Furthermore, another *de novo* case of heterozygous N160S and heterozygous G191R, a previously unreported missense variant within the third ZF have been reported [25] (the study of G191R case is *in press* in Journal of Clinical Immunology). To date, 18 patients, from 7 families with 5 distinct pathogenic AIOLOS variants, including one asymptomatic carrier have been described in the literature. AIOLOS-associated IEI demonstrates relatively high clinical penetrance, with only a single documented asymptomatic carrier. However, it is important to acknowledge the substantial variability in phenotypes and severity, even among patients harboring identical variants.

Notably, the AIOLOS^E82K^ missense variant has been commonly found in general population, exhibiting a minor allele frequency of 1.4e-4 (~ 1 in 7000 individuals, rs369340496). Hence, it is crucial to investigate whether individuals carrying the AIOLOS^E82K^ variant also manifest immune abnormalities associated with AIOLOS haploinsufficiency, or if the reported patients harbor additional genetic predispositions that modify the disease phenotype.

## Clinical Features of AIOLOS-Associated IEI

### Susceptibility to Infections

**Fig. 2:**
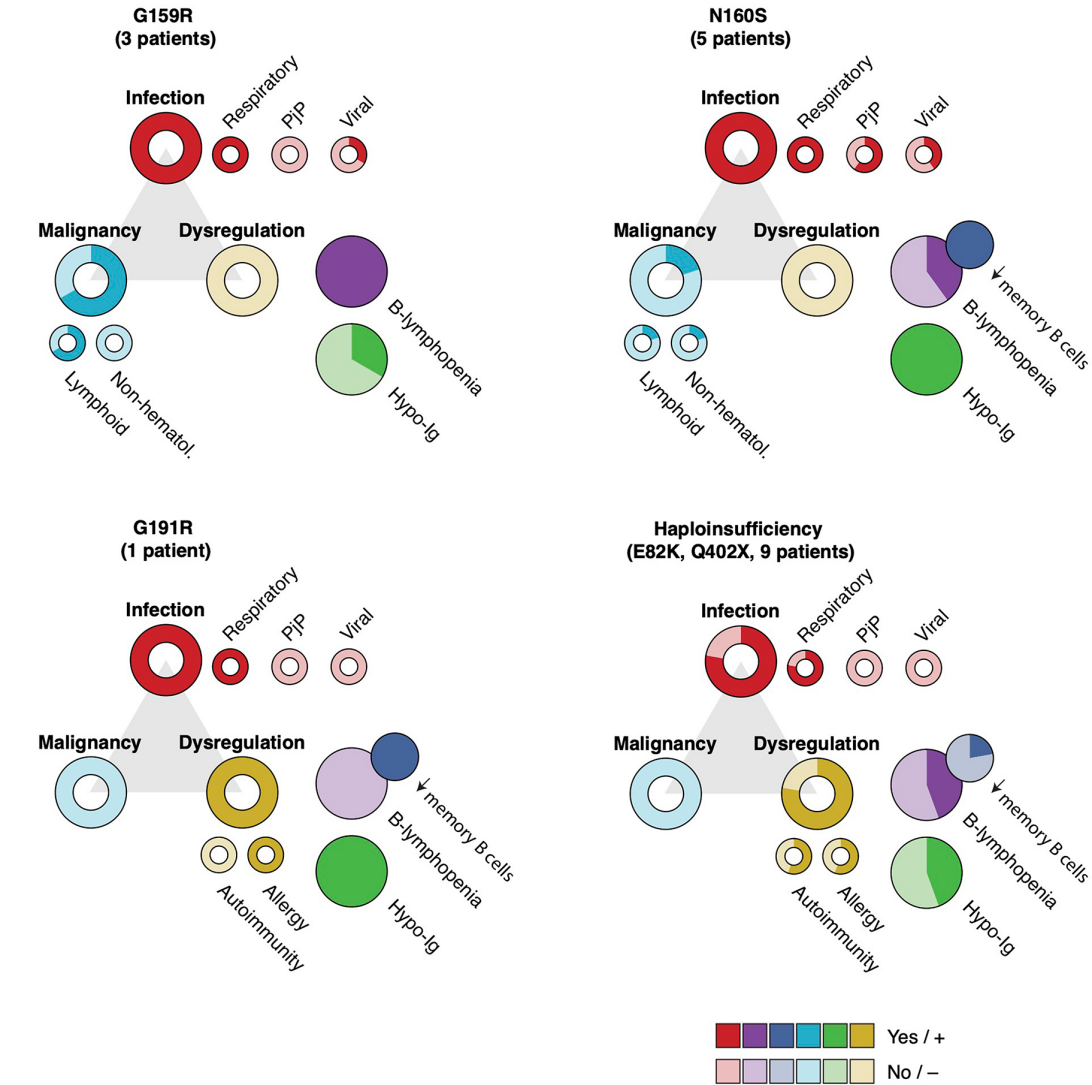
Summary of the clinical and immunological phenotypes of the reported AIOLOS-associated IEI cases. The frequency of clinical manifestations and immunological abnormalities observed in individuals carrying each AIOLOS variant is summarized. Hypo-Ig, hypoimmunoglobulinemia; Non-hematol, non-hematological; PjP, *Pneumocystis jirovecii* pneumonia

Recurrent sinopulmonary infections are the primary infectious complication of AIOLOS-associated IEI, observed in 15/18 patients reported (Fig. 2; Table 1). Recurrent respiratory infections in individuals with AIOLOS^N160S^ (2/5 patients) and AIOLOS^E82K^ (1/6 patients) subsequently resulted in bronchiectasis. The pathogens identified as causative agents of pneumonia in AIOLOS-associated IEI patients include mycobacteria (*Mycobacterium avium* complex and *Mycobacterium tuberculosis*), *Pseudomonas aeruginosa*, *Escherichia coli*, *Acinetobacter baumannii*, *Klebsiella pneumoniae*, *Stenotrophomonas maltophilia*, and *Mycoplasma*. Bacterial infections other than pneumonia in AIOLOS-associated IEI included perianal abscesses, bacterial meningitis, sepsis, urinary tract infection, recurrent streptococcal pharyngitis associated with scarlet fever, and soft tissue infection. As for viral infections, a patient with the AIOLOS^G159R^ exhibited susceptibility to Epstein–Barr virus (EBV) infection, leading to recurrent infectious mononucleosis, EBV-associated hemophagocytic syndrome, and chronic active EBV infection (CAEBV).

**Table 1:** Summary of clinical manifestations in AIOLOS-associated IEI cases

	G159R	N160S	G191R	Q402X	E82K
	3 patients, 1 family	5 patients, 2 families	1 patient, 1 family (*de novo*)	3 patients, 1 family	6 patients, 2 families
**Infections**					
Recurrent sinopulmonary infections	2/3	5/5	1/1	2/3	5/6
Identified pathogens of respiratory infections		MAC, *P. aeruginosa, E. coli, M. tuberculosis*	*A. baumannii, K. pneumoniae, S. maltophilia, Mycoplasma, P. aeruginosa*		
Bacterial infections	Perianal abscess (1/3)	Sepsis (1/5), meningitis (1/5)	*Pseudomonas* sepsis	Urinary tract infection (1/3)	Recurrent streptococcus pharyngitis and scarlet fever (1/6)
Fungal infections		*P, jirovecii pneumonia* (3/5)			
Viral infections	Recurent EBV-IM, recurrent EBV-HLH, CAEBV (1/3)	Cutaneous warts (1/5), HBV, measles, and varicella (1/5)			Persistent herpes zoster (1/6)
Other / non-specified			Upper-limb soft tissue infections, fungal infections	Bilateral submandibular lymphadenitis (1/3)	
**Immune dysregulation**					
Autoimmunity				Immune thrombocytopenia (1/3),	Hashimoto’s thyroiditis (3/6), SLE and autoimmune hepatitis (1/6)
Allergy			Atopic dermatitis	Food allergy (1/3)	Asthma (3/6), atopic dermatitis (2/6), environmental allergy (2/6)
Other / non-specified	Splenomegaly (1/3)	Oligoarticular arthritis (1/5)	Colitis		Erythroderma nodosum and sarcoidosis (1/6), Polymyalgia reumatica and colitis (1/6)
**Malignancy**					
Hematological	B-lymphoma (EBV-associated, 2/3)	CLL (1/5)			
Non-hematological		Metastatic melanoma (1/5)			

CAEBV, chronic active EBV infection; CLL, chronic lymphocytic leukemia; EBV, Epstein–Barr virus; HBV, hepatitis B virus; HLH, hemophagocytic lymphohistiocytosis; IM, infectious mononucleosis; MAC, *Mycobacterium avium* complex; SLE, systemic lupus erythematosus

Viral infections observed in patients with the AIOLOS^N160S^ variant include extensive cutaneous warts, measles, varicella, and persistent hepatitis B virus viremia. Persistent herpes zoster has been documented in a patient with AIOLOS^E82K^. It is of note that *Pneumocystis jirovecii* pneumonia (PjP) has been frequently observed in patients with the AIOLOS^N160S^ variant (3/5 patients), whereas patients with other variants did not contract PjP.

### Immune Dysregulation

AIOLOS haploinsufficiency patients bearing AIOLOS^E82K^ (6 patients) and AIOLOS^Q402X^ (3 patients) exhibit diverse manifestations of autoimmunity, allergy and other forms of immune dysregulation (Fig. 2; Table 1). Overall, immune dysregulation-related phenotypes were observed in 7 out of 9 patients with haploinsufficient variants. Autoimmune diseases in AIOLOS haploinsufficiency (5/9 patients) include Hashimoto’s thyroiditis, immune thrombocytopenia, SLE and autoimmune hepatitis. Allergic diseases, such as asthma, atopic dermatitis, food allergy, and environmental allergy are prevalent among AIOLOS haploinsufficiency patients (5/9 patients). Other manifestations potentially associated with immune dysregulation in AIOLOS haploinsufficiency include erythema nodosum and sarcoidosis in one patient, and polymyalgia rheumatica and colitis in another patient. Additionally, the patient with AIOLOS^G191R^ variant presented with atopic dermatitis and colitis.

Contrastingly, patients with AIOLOS^G159R^ (3 patients) and AIOLOS^N160S^ (5 patients) variants did not show any evidence of autoimmunity. Nonetheless, it is noteworthy that one patient with the AIOLOS^G159R^ variant presented with splenomegaly and pancytopenia. Additionally, one patient with the AIOLOS^N160S^ variant experienced oligoarticular arthritis; however, neither condition was confirmed as an autoimmune disease.

### Malignancy

Lymphoid malignancies have been reported in individuals carrying the AIOLOS^G159R^ (2/3 patients) and AIOLOS^N160S^ (1/5 patients) variants (Fig. 2; Table 1). One patient with the AIOLOS^G159R^ variant developed a mixture of follicular lymphoma and EBV-positive diffuse large B cell lymphoma (DLBCL), while another patient with the AIOLOS^G159R^ variant developed EBV-DLBCL. In contrast, one patient with the AIOLOS^N160S^ variant developed chronic lymphocytic leukemia (CLL) and metastatic melanoma. Other than these cases, no malignancies have been observed in the patients with AIOLOS-associated IEI.

### Immunological Examinations

The AIOLOS^G159R^ variant was associated with profound B-lymphopenia in the peripheral blood (1–3% of lymphocytes). Bone marrow examination of a patient with this variant revealed a reduction in B-lineage-committed progenitors. B-lymphopenia and dysgammaglobulinemia were commonly observed in patients with the AIOLOS^G159R^, with increased IgA levels observed in all patients, while decreased IgG was only observed in 1 patient. B-lymphopenia was observed in 2 patients with AIOLOS^N160S^, while the remaining patients exhibited normal to high B cell counts (3/5 patients). Despite normal B cell numbers in the peripheral blood, panhypoimmunoglobulinemia is a common feature of patients with the AIOLOS^N160S^. B cells from the patients are characterized by low CD21 expression and minimal levels of CD23. Other B cell phenotypes associated with AIOLOS^N160S^ include nearly absent memory B cells and a defective differentiation capacity of B cells into plasmablasts under in vitro differentiation conditions with CD40L and IL-21 stimulation. Patient-derived B cells showed decreased proliferation upon stimulation with CD40L and anti-IgM, IL-4, or IL-21. Additionally, the B cells showed skewed κ and λ light chain expression in 2 patients; and decreased expression of IgD, IgM, and κ and λ light chains was observed in all the affected individuals [23]. Despite these CD40 signaling defects observed, patients with the AIOLOS^N160S^ variant did not show hyper-IgM phenotypes, probably because of the intrinsic B cell defects. Another case with AIOLOS^N160S^ variant displayed a decreased expression of IgD in B cells, although the expression level of IgM was not decreased compared to the controls [25]. Four out of 9 patients with AIOLOS haploinsufficiency exhibited B-lymphopenia, and 2 patients showed decreased memory B cells. Low IgG level was observed in 4 out of 9 patients. In contrast to individuals with the AIOLOS^N160S^ variant, B cells in AIOLOS haploinsufficiency patients showed normal proliferation and differentiation into plasmablasts. The B cells stimulated with CD40L and IL-21 had normal production of IgM, but showed reduced production of IgG and/or IgA (8/9 patients). In the patient harboring AIOLOS^G191R^ variant, B cell number was not decreased but there was a reduction in memory B cells accompanied by the decrease in serum levels of IgG, IgM, and IgA.

AIOLOS-associated IEI also results in T cell abnormalities. In a patient with AIOLOS^G159R^, CD4^+^ T cells were skewed toward a memory phenotype, whereas T cells displayed an activated phenotype, along with an imbalanced Th subset with a decreased proportion of Th17 cells and an increased proportion of Th1* cells. The intensity of the surface expression of CD3 and T cell receptor (TCR)-α/β was decreased. In patients harbouring the AIOLOS^N160S^ variant, a relatively increased fraction of recent thymic emigrant (RTE) naïve T cells was observed, accompanied by a decrease in the memory T cells and follicular helper T cells (Tfh) within the majority of subjects (4/4 patient tested). Marked reduction in Th1 cells, but not Th2 or Th17 cells, was observed in all cases. CD40L expression on activated T cells was markedly decreased following PMA and ionomycin stimulation in all the patients. The subsequent report of a patient with AIOLOS^N160S^ demonstrated no apparent skewing toward naïve T cells or notable increase in the RTE fraction. T-lymphopenia was observed in a patient with AIOLOS haploinsufficiency (1/9 patient) and a patient harboring AIOLOS^G191R^ variant. In the patient with AIOLOS^G191R^ variant, there were reductions in naïve CD4^+^ and CD8^+^ T cells. Similarly, decreased levels of naïve CD4^+^ T cells and RTE were also observed in 2 patients with AIOLOS^Q402X^ variant (2/3 patients), suggesting T lymphopoiesis is also affected in the AIOLOS-associated IEI.

A notable reduction in the number of NK cells was observed in 1 patient carrying the AIOLOS^G159R^ variant and 1 patient in her 70s with AIOLOS^E82K^ variant (1/6 patient). Given the association between AIOLOS degradation and impaired ILC-1 development, future studies should focus on elucidating the development of ILCs in patients with AIOLOS-associated IEI.

### In vitro Characterization of IEI-Causing AIOLOS Variants

AIOLOS^G159R^ was identified as a loss-of-function (LOF) variants based on DNA binding to the consensus-binding sequence of AIOLOS. AIOLOS^G159R^ variant not only lost binding to the AIOLOS consensus-binding motif but also gained affinity to aberrant sequences [22]. Another in vitro functional assay to assess the ability of IKZF to target pericentromeric heterochromatin (PC-HC) indicated that AIOLOS^G159R^ could not target PC-HC, consistent with a LOF variant. Additionally, transcriptional activity of AIOLOS^G159R^ was impaired when compared to wildtype AIOLOS. Interestingly, when expressed with IKAROS, AIOLOS^G159R^ interfered with the PC-HC-targeting ability and the transcriptional repressive activity of IKAROS, suggesting that AIOLOS^G159R^ acts in a dominant-negative manner against IKAROS.

AIOLOS^N160S^ is also a LOF variant [23]. However, in contrast to AIOLOS^G159R^, AIOLOS^N160S^ did not exert a dominant-negative effect against IKAROS in the PC-HC targeting assay; however, it acted negative-dominance against wild-type AIOLOS for DNA binding and PC-HC targeting. Genome-wide binding of AIOLOS assessed in in vitro-expanded T cells with the AIOLOS^N160S^ showed differences compared to that assessed in T cells derived from healthy controls, however, the binding sequences of AIOLOS remained unchanged.

The AIOLOS^G191R^ represents a missense variant occurring in the third ZF domain of AIOLOS. The glycine at position 191 is recognized as crucial for DNA interaction and ZF functionality. Indeed, AIOLOS^G191R^ variant loses DNA binding ability, exerting dominant-negative effect against wildtype AIOLOS. Also, AIOLOS^G191R^ led to the compromised stability of the protein.

Interestingly, pathogenic missense variants within the DNA-binding ZF of AIOLOS, specifically G159R, N160S, and G191R, have been shown to exert a dominant-negative effect on wildtype AIOLOS. This finding is in contrast to the majority of IEI-causing IKAROS variants within the DNA-binding ZF, which do not display a dominant-negative effect on wildtype IKAROS. While the precise mechanism is yet to be uncovered, the dominant-negative behavior of these AIOLOS variants could be attributed to the wildtype AIOLOS’s relatively weaker binding affinity to its consensus motif compared to IKAROS. This difference may make wildtype AIOLOS more susceptible to interference from non-functional mutants via dimerization. Understanding the molecular mechanism that distinguishes these variants, in terms of how they alter AIOLOS’s function and potentially interfere with the function of IKAROS and other molecules, is crucial for unraveling the diverse immune abnormalities triggered by these distinct variants. This area will be a key focus for future research, aiming to understand how germline variants in the *IKZF3* gene result in diverse immune abnormalities.

Currently identified pathogenic AIOLOS haploinsufficient variants include the truncating Q402X and the E82K missense variants [24]. AIOLOS^E82K^ mutant protein showed high proteosomal degradation and compromised protein stability. AIOLOS^Q402X^ lacks C-terminal dimerization domain, failed to target PC-HC, but bound to AIOLOS consensus sequence as monomers. Intriguingly, despite lacking the canonical dimerization domain, AIOLOS^Q402X^ demonstrated dimerization capacity against wildtype AIOLOS but not with other IKZF molecules, through ZF1. In addition, AIOLOS^Q402X^ exhibited a notable absence of post-translational modification via small ubiquitin-related modifier (SUMOylation) and impaired interaction with SUMO1 or SUMO2.

### Aiolos-mutant Mouse Models

The complexity of understanding AIOLOS-associated IEI arises from the disparity between the phenotypes observed in Aiolos-deficient model organisms and patients with AIOLOS-associated IEI. While Aiolos-null mice showed a minimal impact on early B cell development and elevated immunoglobulin levels [9], patients with AIOLOS-associated IEI exhibited profound B-lymphopenia (G159R) and marked hypoimmunoglobulinemia (N160S). Although both missense variants were confirmed as LOF through in vitro experiments, the loss of Aiolos in murine models failed to replicate patient phenotypes. Is it because Aiolos plays different roles in murine and human lymphocyte development? Or is it because the expression of dysfunctional AIOLOS is pathogenic through dominant-negative and/or neomorphic mechanisms? Using CRISPR/Cas9 technology, we created knock-in mouse models, specifically introducing AIOLOS^G159R^ and AIOLOS^N160S^ variants (equivalent to Aiolos^G158R^ and Aiolos^N159S^ in the murine *Ikzf3* gene), which were further investigated (Table 2) [22, 23, 26].

**Table 2:** Summary of immune abnormalities in Aiolos-knockout and AIOLOS-associated IEI mouse models

	*Ikzf3* ^*–/–*^	*Ikzf3* ^*G158R/+*^	*Ikzf3* ^*G158R/G158R*^	*Ikzf3* ^*N159S/+*^	*Ikzf3* ^*N159S/N159S*^
**B cell**	**B cell progenitors**				
	Partial differentiation blockade at pre-B cells	Differentiation blockade at pre-pro B cell to pre-B cell		Not evident	↑ immature B
	**Mature B**				
	**BM**				
	↓ recirculating B cells	↓ recirculating B cells	↓↓ recirculating B cells	↑ recirculating B cells	↓ recirculating B cells
	↓ long-lived plasma cells				
	**Periphery**				
	No ↓ in spleen	↓ Splenic B, progressive decrease	↓↓ Splenic B	↑ B cell in PB, no ↑ in spleen	↓ B cell in PB, no ↓ in spleen
	↓ IgM^hi^IgD^–^ B, ↓ MZ B	↓ FO B, ↓ MZ B, ↓ GC B (↓ CD21 and CD23 expression)		↓ FO B, ↓ MZ B (↓ CD21 and ↓↓ CD23 expression)	
	Activated phenotype			↓ IgD expression	↓↓ IgD expression
	Spontaneous GC formation			↓ CD62L expression	
	**Other**				
	↓↓ B1a cells in peritoneal cavity			↓ B1a and ↑ B2 cells in peritoneal cavity	↓ B1a and B2 cells in peritoneal cavity
	↑ serum IgG/E				↓ B lineage in Peyer’s patches (GC B cells were relatively spared)
	Production of autoantibodies			↓ serum IgA/M in aged mice	↓ serum IgG/A/M
**T cell**	**Thymocytes**				
	Not affected	↓ TCRβ expression in post-selection thymocytes		↓ CD62L expresion in DP and later stages of T cells	
			↑ CD4^lo^CD8^+^ thymocytes		
			↓ CD4SP		
	**Periphery**				
	↑ proliferation and IFN-γ/IL-2 production in response to TCR stimulation	↓ CD3ε and TCRβ expression		↑ T cell in PB, no ↑ in spleen	
	Defective Th17 differentiation		CD4^+^ T skewing to memory	CD4/CD8, naive/memory comparable in PB	↑ CD4^+^ T and ↓ naive T cells in PB
		↓ CD8^+^T cell			CD8^+^ T cells memory-skewing in PB
		↑ DN T			Nearly absent Tfh in Peyer’s patches
**Other hematopoietic cell lineages**	↓ terminally matured NK cells	Not evaluated		Comparable Neu and DC	Comparable Neu, minimally decreased DC
				↓ Eo, ↓ NK in spleen	
**Malignancy**	B and T cell lymphoma in aging mice	Not observed		Not observed	

BM, bone marrow; DC, dendritic cells; DN, double-negative; DP, double-positive; Eo, eosinophils; FO, follicular; GC, germinal center; LN, lymph nodes; MZ, marginal zone; Neu, neutrophils; NK, natural killer cells; SP, single-positive; TCR, T cell receptor; Tfh, follicular helper T cells; PB, peripheral blood

#### Aiolos-Null Mouse

B cell defects in Aiolos-knockout mice are characterized by a partial developmental blockade at the pre-B cell stage and an abnormal activation status in peripheral B cells [9]. While the number of B-lineage cells was unaffected, Aiolos-knockout mice presented an accumulation of B cell progenitors (pro-B and pre-B cells) and a decrease in the number of mature naïve B cells and recirculating B cells in the bone marrow. In the periphery, splenic B cells were normal in number, but there was a decrease in the number of newly exported naïve B cells and marginal zone (MZ) B cells. Splenic B cells mainly exhibited follicular (FO) phenotypes and were activated. Spontaneous germinal center (GC) formation without immunization was observed in the mice. The proportion of long-lived high-affinity plasma cells in the bone marrow is significantly reduced in Aiolos-knockout mice [27]. Increased levels of serum IgG and IgE and autoantibodies are also frequently detected [9]. In contrast to conventional B2 cells, the proportion of peritoneal B1a cells is markedly reduced in Aiolos-knockout mice.

T cell development was unaffected; however, thymocytes and peripheral T cells showed enhanced proliferation following TCR stimulation, and there was an increase in IFN-γ and IL-2 production after TCR and CD28 stimulation [9]. Aiolos-deficient CD4^+^ T cells exhibit defective Th17 differentiation [11]. Aiolos-knockout mice also exhibit defective NK cell maturation. The proportion of terminally mature NK cells is decreased in Aiolos-knockout mice, and these NK cells show hyperresponsiveness to activation and proliferation in response to IL-15 stimulation [17].

Aging Aiolos-deficient mice also develop splenomegaly due to the expansion of the B cell compartment. Finally, aging Aiolos-knockout mice develop lymphoma of both B and T cell origins [9].

#### Aiolos^G158R^ Mouse

The most prominent immune defects in the Aiolos^G158R^ mouse model were observed during early B cell differentiation, closely resembling the observations made in patients with the corresponding variant [22]. Developmental blockage at the pre-pro-B cell to pro-B cell and pre-B cell stages was observed in both heterozygous and homozygous Aiolos^G158R^ mice. Transcriptomic analysis of heterozygous Aiolos^G158R^ pre-B cells revealed decreased expression of genes associated with B cell differentiation. Notably, the dysregulated genes contained a high proportion of putative target genes regulated by Ikaros, as suggested by the binding of Ikaros at the transcription start site in pre-B cells. This impairment of B cell differentiation was further reflected by a marked reduction in the number of splenic B cells. Additionally, a gradual but more profound B cell lymphopenia was observed in Aiolos^G158R^ mice with aging. B cell developmental defects in Aiolos^G158R^ mice were cell-intrinsic. Furthermore, CD21 and CD23 expression was downregulated in splenic B cells. Similar to those observed in human patients, the expression levels of CD3ε and TCRβ were reduced in the T cells of both homozygous and heterozygous Aiolos^G158R^ mice. Other T cell phenotypes in Aiolos^G158R^ mice include a decrease in the CD8^+^ fraction of lymph node T cells and the emergence of CD4^–^CD8^–^ T cells. CD4^+^ T cells were skewed towards memory phenotypes in homozygous mice.

The genome-wide binding patterns and sequences of Ikaros and Aiolos were altered in thymocytes of homozygous Aiolos^G158R^ mice, suggesting Aiolos^G158R^ exerts its pathogenicity by interfering with the function of Ikaros via heterodimer formation. Furthermore, a new mutant mouse line was generated by introducing a frameshift mutation in the C-terminus of Aiolos^G158R^ mutant allele, and a C-terminal ZF deletion in this Aiolos^G158R:Δc−ZF^ mouse line rescued the B cell differentiation defects and reduced CD3ε and TCRβ expression observed in Aiolos^G158R^ mice.

#### Aiolos^N159S^ Mouse

The early B cell developmental defects observed in Aiolos^G158R^ mice were not evident in Aiolos^N159S^ mice [23, 26]. B cell numbers were variously affected in the bone marrow and periphery of the heterozygous and homozygous Aiolos^N159S^ mice. Bone marrow competition assay revealed that the B cell generation ability of these mice was inferior to that of the wildtype. In addition, there was decreased expression of CD21 and minimal expression of CD23 in the peripheral B cells of both heterozygous and homozygous Aiolos^N159S^ mice. The surface expression of IgD was markedly decreased in Aiolos^N159S^ mice. In the Peyer’s patches, the proportion of B-lineage cells was markedly decreased in homozygous Aiolos^N159S^ mice. The number of B1a cells in the peritoneal cavity in homozygous Aiolos^N159S^ mice was also markedly reduced. Like patients with the AIOLOS^N160S^ variant, the serum levels of IgG, IgA, and IgM of homozygous Aiolos^N159S^ mice were also decreased.

The T cell fraction in the peripheral blood leukocytes of Aiolos^N159S^ mice increased in a gene dose-dependent manner. Specifically, there was an increased fraction of CD4^+^ T cells and decreased fractions of naïve CD4^+^ and CD8^+^ T cells in the peripheral blood of homozygous Aiolos^N159S^ mice. Additionally, the proportion of Tfh cells was markedly decreased in the Peyer’s patches of homozygous mice.

One hallmark phenotype observed in the T and B cells of Aiolos^N159S^ mice is the downregulation of CD62L, encoded by the putative Ikaros target gene *Sell* [28]. CD62L mediates lymphocyte homing to secondary lymphoid organs [29]. Indeed, the homing ability of T and B cells in the lymph nodes was abrogated in homozygous Aiolos^N159S^ mice [26]. The overexpression of Ikaros, but not Aiolos, in homozygous Aiolos^N159S^ B cells restored CD62L expression. Although the precise mechanism behind CD62L downregulation in Aiolos^N159S^ mice remains unclear and will be the subject of future studies, the fact that this phenotype can be compensated by the overexpression of Ikaros, but not Aiolos, suggests the following possibilities. First, Aiolos^N159S^ may negatively affect the function of Ikaros in a physiological context. Alternatively, the downregulation of CD62L could result from a neomorphic function of Aiolos^N159S^, with compensation achievable only through an overabundance of Ikaros. Additionally, Ikaros and Aiolos may regulate CD62L expression through different molecular mechanisms, and the failure to induce CD62L by Aiolos^N159S^ could be compensated by the overexpression of Ikaros.

In addition to T and B cells, the proportions of splenic eosinophils and NK cells decreased in both heterozygous and homozygous Aiolos^N159S^ mice.

### AIOLOS Mutations in Cancer

The involvement of AIOLOS in various cancers has been well-documented. For instance, the deletion of the *IKZF3* gene has been reported in pediatric patients with B-acute lymphoblastic leukemia (ALL) [30]. Additionally, somatic mutations in *IKZF3* have been observed in Waldenström macroglobulinemia [31] and non-Hodgkin’s lymphoma [32], while the somatic L162R missense mutation has been commonly identified in CLL [33]. In a murine model harboring the hotspot *IKZF3* missense mutation in CLL, it was observed that the mutant AIOLOS triggered the activation of the BCR and nuclear factor-κB (NF-κΒ) signaling pathways, mirroring the effects of *IKZF3* overexpression in primary CLL cells [34]. Furthermore, the aberrant overexpression of AIOLOS has been associated with poor prognosis in CLL [35–37]. The upregulation of AIOLOS has also been documented in follicular cell lymphoma [38]. Collectively, these findings indicate that both the loss and gain of AIOLOS function can contribute to the development of lymphoid malignancies.

### Homologous Variants in IKZF Proteins: Are they Related?

Germline monoallelic and biallelic LOF variants in the *IKZF2* gene, which encodes HELIOS, cause IEIs predominantly presenting with autoimmunity and immunodeficiency [39–41]. Very recently, another HELIOS-associated IEI with distinct phenotypes was reported, which was caused by heterozygous dominant-negative variants of HELIOS: HELIOS^G153R^ and an exon 5 duplication [42]. HELIOS^G153^ is located in the second ZF of HELIOS and is homologous to AIOLOS^G159^. Clinically, the patients with present syndromic features of craniofacial differences, congenital athelia, sensorineural hearing loss, cleft palate, and developmental delays. Immunologically, T lymphopenia with low TCR excision circles was also observed. Interestingly, patients with HELIOS^G153R^ but not those with exon 5 duplication presented with B-lymphopenia associated with a poor vaccine response and mildly decreased IgG levels, a phenotype reminiscent of AIOLOS-associated IEI caused by AIOLOS^G159R^ variant. Some immune abnormalities caused by HELIOS^G153R^ may have resulted from the dysfunction of other IKZF members, as observed in the AIOLOS^G159R^ variant.

The homologous variant AIOLOS^N160S^ in IKAROS (N159S) causes the most severe form of IKAROS deficiency [43–47]. Clinically, patients with IKAROS N159 missense variants present opportunistic infections, including PjP, and severe bacterial, viral, fungal, and parasitic infections. Patients with IKAROS^N159S/T^ present with severe B-lymphopenia and hypoimmunoglobulinemia. Their T cells were skewed to naïve phenotypes, and their memory T cell proportions were markedly reduced. The IKAROS^N159S/T^ variant affects other hematopoietic cell lineages and decreases the numbers of neutrophils, eosinophils, NK cells, and DCs in a subset of patients. IKAROS^N159S/T^ variants exerted dominant-negative effects against wild-type IKAROS and AIOLOS. The striking clinical and immunological similarities between patients with AIOLOS^N160S^ and IKAROS^N159S/T^ variants may suggest a common pathogenic mechanism between these two diseases.

Heterozygous truncating variants preserving ZF1-3 of IKAROS are classified as dimerization defect/haploinsuffiency variants in IKAROS-associated IEI [24, 48]. Notably, similarities exist between AIOLOS haploinsufficiency and IKAROS dimerization defect. Clinically, both conditions are predominantly associated with autoimmune diseases, along with a relatively milder infectious complications compared to other variants. Patients with IKAROS dimerization defect variants typically manifest progressive B-lymphopenia (~ 40%) and hypoimmunoglobulinemia (70%), and occasionally develop lymphoid malignancies. In contrast, these characteristics are less pronounced in AIOLOS haploinsufficiency patients, with no observed occurrences of malignancy. Heterozygous truncated variants of HELIOS, R291X and Y200X, have been identified in the patients exhibiting immunomodulatory abnormalities, with the latter additionally associated with a combined immunodeficiency phenotype [39, 41]. Interestingly, reduction in SUMOylation was noted in C-terminal truncating variants of IKZF proteins such as IKAROS, AIOLOS, and HELIOS [24, 41, 48].

### Unanswered Questions

The molecular mechanisms underlying AIOLOS-associated IEI remain elusive. Both AIOLOS^G159R^ and AIOLOS^N160S^ variants represent LOF with respect to DNA binding to the canonical binding sequence and transcriptional activity. Another missense variant in ZF3, G191R, is likewise demonstrated to be LOF. AIOLOS^G159R^ exhibited a dominant-negative effect against AIOLOS and IKAROS, while AIOLOS^N160S^ exerted this effect exclusively against AIOLOS. Notably, the normal function of Ikaros was perturbed in murine models with the Aiolos^G158R^ mutation, as evidenced by the ChIP-seq of thymocytes and transcriptomic analysis of pre-B cells. In Aiolos^N159S^ mice, impaired lymphocyte homing to the lymph nodes and downregulation of CD62L in T and B cells can be attributed, at least in part, to the inhibition of Ikaros function. How do these adjacent missense variants give rise to distinct abnormalities in the human and murine immune systems? As exemplified by the newly acquired affinity of the AIOLOS^G159R^ and Aiolos^G158R^ variants for aberrant DNA sequences, the neomorphic properties of the AIOLOS^G159R^ and AIOLOS^N160S^ variants could potentially explain these divergent characteristics. It is also plausible that the proteins interacting with these two variants differ substantially. Although it has not been investigated, exploring whether AIOLOS^G191R^ exerts a dominant negative effect against AIOLOS or other IKZF molecules presents as an intriguing subject for further investigation.

Furthermore, an optimal therapeutic approach for AIOLOS-associated IEI remains undetermined. Considering the recurrent infectious complications prevalent among patients with AIOLOS-associated IEI, it is advisable to consider immunoglobulin replacement therapy and prophylactic antibiotics, including PjP prophylaxis, for individuals with the AIOLOS^N160S^ variant. Within patients with the AIOLOS^G159R^ variant, hematopoietic cell transplantation (HCT) was performed in those with lymphoma. The exploration of optimal indications and timing for HCT will be essential in refining clinical practices for the patients with AIOLOS-associated IEI. The immune dysregulation observed in patients with AIOLOS^E82K^ variant was managed with topical and/or systemic immunosuppressants, leading to varied responses. Treatment with proteasome inhibitors restored the expression of AIOLOS haploinsufficient variants, especially AIOLOS^E82K^, in in vitro experiments using cell lines, thereby highlighting the proteasome inhibitors as a potential therapeutic option. However, it remains unclear how the restoration of truncated AIOLOS, lacking the C-terminal domain, influences lymphocyte differentiation and function, and this should be the subject for the future studies. Furthermore, in the future, the utilization of gene therapy stands as a promising strategy for treating AIOLOS-associated IEI. Accumulating evidence indicates distinct molecular mechanisms for AIOLOS haploinsufficiency and specific missense variants such as G159R and N160S. Given these differences, the optimal therapeutic strategy for gene therapy, such as gene addition or gene editing/correction, should be tailored and optimized according to the unique molecular pathogenesis associated with each variant.

## Conclusions

AIOLOS-associated IEI is caused by heterozygous LOF variants in the *IKZF3* gene. To date, five distinct variants have been identified as causative in AIOLOS-associated IEI: E82K, G159R, N160S, G191R, and Q402X. The hallmark features of AIOLOS-associated IEI include recurrent sinopulmonary infections, occasional occurrences of bacterial, viral infection and PjP. Notably, the AIOLOS^N160S^ variant is linked to the most severe form of immunodeficiency, while patients with AIOLOS haploinsufficiency (E82K and Q402X variants) predominantly present with immune dysregulation. Furthermore, a subset of patients has exhibited lymphoid malignancies. Immunologically, approximately half of the patients demonstrate B-lymphopenia and hypoimmunoglobulinemia. Although the underlying molecular mechanisms leading to the divergent immune abnormalities caused by pathogenic AIOLOS variants remain elusive, the perturbation of IKAROS function has been identified as the core molecular pathogenesis caused by AIOLOS^G159R^ variant through murine models. Further studies involving more patients would help us understand the comprehensive clinical spectrum of AIOLOS-associated IEI.

## Data Availability

No datasets were generated or analysed during the current study.
